# Mechanically Resistant Poly(*N*‐vinylcaprolactam) Microgels with Sacrificial Supramolecular Catechin Hydrogen Bonds

**DOI:** 10.1002/advs.202104004

**Published:** 2022-02-20

**Authors:** Emilia Izak‐Nau, Susanne Braun, Andrij Pich, Robert Göstl

**Affiliations:** ^1^ DWI – Leibniz Institute for Interactive Materials Forckenbeckstr. 50 Aachen 52056 Germany; ^2^ Institute of Technical and Macromolecular Chemistry RWTH Aachen University Worringerweg 1 Aachen 52074 Germany; ^3^ Aachen Maastricht Institute for Biobased Materials (AMIBM) Maastricht University Brightlands Chemelot Campus Geleen 6167 RD The Netherlands

**Keywords:** colloids, mechanical properties, microgels, optical force probes, sacrificial bonds, shear force

## Abstract

Microgels (μgels) swiftly undergo structural and functional degradation when they are exposed to shear forces, which potentially limit their applicability in, e.g., biomedicine and engineering. Here, poly(*N*‐vinylcaprolactam) μgels that resist mechanical disruption through supramolecular hydrogen bonds provided by (+)‐catechin hydrate (+C) are synthesized. When +C is added to the microgel structure, an increased resistance against shear force exerted by ultrasonication is observed compared to μgels crosslinked by covalent bonds. While covalently crosslinked μgels degrade already after a few seconds, it is found that μgels having both supramolecular interchain interactions and covalent crosslinks show the highest mechanical durability. By the incorporation of optical force probes, it is found that the covalent bonds of the μgels are not stressed beyond their scission threshold and mechanical energy is dissipated by the force‐induced reversible dissociation of the sacrificial +C bonds for at least 20 min of ultrasonication. Additionally, +C renders the μgels pH‐sensitive and introduces multiresponsivity. The μgels are extensively characterized using Fourier‐transform infrared, Raman and quantitative nuclear magnetic resonance spectroscopy, dynamic light scattering, and cryogenic transmission electron microscopy. These results may serve as blueprint for the preparation of many mechanically durable μgels.

## Graphical abstract

The incorporation of a supramolecular motif provides sacrificial bonds and renders microgel networks remarkably resistant against shear force in solution. Microgels containing both noncovalent motifs and covalent crosslinkers exhibit superior resistance against mechanical damage than those containing only one type alone. The supramolecular interactions render the μgels pH‐sensitive and endow the material with multiresponsivity.

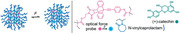

## 1 Introduction

μgels are soft polymeric networks oftentimes designed to respond to external stimuli, such as temperature, pH, light, or redox potential.^[^ ^1^ ^]^ They are widely used for applications in, e.g., drug delivery, cell encapsulation, or separation and purification technologies.^[^ ^2^, ^3^, ^4^, ^5^ ^]^ Poly(*N*‐isopropylacrylamide) (PNIPAAm) and poly(*N*‐vinylcaprolactam) (PVCL) are the two most popular temperature‐responsive polymers used for μgel preparation.^[^ ^5^ ^]^ While the use of PNIPAAm is limited in biomedical applications due to its toxicity,^[^ ^6^ ^]^ PVCL μgels have been shown to be biocompatible retaining the advantageous temperature‐responsive properties of PNIPAAm.^[^ ^6^, ^7^, ^8^ ^]^ Swelling behavior, deformability, and surface activity are strongly influenced by the internal structure of the μgel.^[^ ^9^, ^10^, ^11^ ^]^ The number, spatial distribution, and chemical identity of the crosslinkers connecting the functional main chains control these parameters. While PNIPAAm μgels can be obtained by self‐crosslinking during dispersion polymerization,^[^ ^12^ ^]^ it is necessary to use covalent crosslinkers^[^ ^13^, ^14^ ^]^ or supramolecular motifs^[^ ^3^ ^]^ for the construction of PVCL μgels.

**Figure 1:**
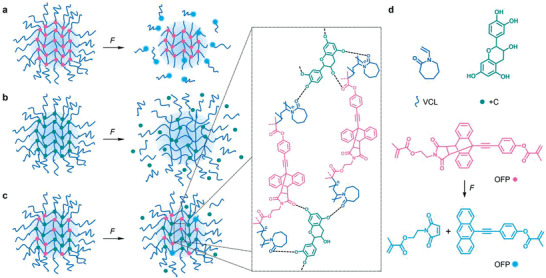
Response of μgels to shear force *F* induced by ultrasonication in dispersion containing a) exclusively covalent crosslinks, b) exclusively supramolecular interchain interactions, or c) both covalent crosslinks and supramolecular interchain interactions. d) Chemical structures of the molecular components of the μgels.

However, structuring these functionally useful macromolecules into hundreds of nm large polymeric networks comes at the price of a very low resistance against mechanical shear force in solution. So far the mechanical properties of μgels have been mainly studied either using rheology or atomic force microscopy (AFM).^[^ ^15^, ^16^, ^17^, ^18^, ^19^ ^]^ While rheological studies provide insight into the viscoelastic behavior of the bulk dispersion,^[^ ^20^, ^21^ ^]^ AFM investigates the local gel topology and the stimulation‐induced modulation of the mechanical properties of surface‐anchored μgels.^[^ ^22^, ^23^, ^24^ ^]^ However, shear‐induced molecular transformations during the shear process in dispersion have not been investigated. We have recently shown that covalently crosslinked PVCL μgels rapidly undergo shear force‐induced degradation by shearing with an ultrasonic processor (shear rate $\dot{\gamma }$ ≈ 10^9^ s^−1^) , ^[^ ^25^, ^26^ ^]^ a mechanical disperser ($\dot{\gamma }$ ≈ 10^6^ s^−1^),^[^ ^25^ ^]^ or a simple hand‐induced extrusion through a needle ($\dot{\gamma }$ ≈ 10^5^ s^−1^)^[^ ^27^ ^]^ accompanied by the loss of their structural functionality (**Figure** **1**a).^[^ ^28^ ^]^ Clearly, this sets back μgel processability as well as applications in flowing media in which shear forces are expected, for example, caused by extrusion, injection, or filtration processes.^[^ ^28^ ^]^ Especially when suggesting μgels as drug delivery systems the circulation within the bloodstream of living organisms is a practical example and illustrates the need to develop a solution to this problem.

The toughening of bulk hydrogel networks has been achieved by the introduction of topological features, such as molecular weaving^[^ ^29^ ^]^ and slide‐ring crosslinks,^[^ ^30^, ^31^ ^]^ or by equipping the material with sacrificial bonds.^[^ ^32^, ^33^, ^34^, ^35^, ^36^, ^37^ ^]^ Yet, such concepts have, to the best of our knowledge, never been implemented to improve the mechanical properties of μgels.

Here, we incorporate dynamic supramolecular interchain interactions promoted by +C into PVCL μgels and thereby endow them with a significantly increased resistance against mechanical degradation (Figure 1b). The comparatively weak but reversible noncovalent interactions act as sacrificial bonds and dissipate mechanical energy on the molecular level by breaking before the covalent polymer backbone undergoes irreversible structural failure.^[^ ^38^ ^]^ +C binds the PVCL backbone by hydrogen bond (H‐bond) interactions between the phenolic groups of +C and the carbonyl groups of PVCL.^[^ ^39^ ^]^ In addition, hydrophobic interactions between the lactam rings of PVCL and the aromatic groups of +C contribute to this.^[^ ^40^ ^]^ Since catechins are polyphenolic flavonoids,^[^ ^41^, ^42^ ^]^ they exhibit biocompatible and antioxidant properties rendering them suitable for biomedical applications.^[^ ^42^, ^43^, ^44^, ^45^ ^]^ Alongside, optical force probes (OFPs)^[^ ^46^, ^47^, ^48^ ^]^ based on the Diels‐Alder adduct of a 9‐*π*‐extended anthracene and maleimide^[^ ^49^, ^50^ ^]^ are incorporated as covalent crosslinkers into the μgels through methacrylate groups (Figure 1c). While providing bifunctional crosslinking comparable to *N,N'*‐methylenebisacrylamide (BIS), the OFP is transformed into a highly fluorescent state upon force‐induced covalent bond scission enabling the observation of stress concentrations with high local resolution.^[^ ^51^, ^52^ ^]^ The presence or absence of mechanical energy dissipation through the sacrificial +C bonds is thus visualized by the absence or presence of a fluorescence signal. Additionally, Fourier‐transform infrared (FTIR), quantitative Raman, and quantitative nuclear magnetic resonance spectroscopy, dynamic light scattering (DLS), electrophoretic mobility (*μ*_e_), and cryogenic transmission electron microscopy (cryoTEM) are employed to characterize the μgels.

## 2 Results and Discussion

### 2.1 Synthesis and Characterization of the μgels

PVCL‐based μgels bonded only with +C and those with both +C and OFP were synthesized by dispersion polymerization in water using 2,2'‐azobis(2‐methylpropionamidine)dihydrochloride (AMPA) as initiator and cetyltrimethylammonium bromide (CTAB) as surfactant. Three different batches of μgels, varied in feed concentration of +C (5, 10, and 15 mol%), were prepared. +C concentrations lower than 5 mol% in the reactant feed led to rapid agglomeration of the μgels likely caused by +C oxidation.^[^ ^53^ ^]^ Because of its insolubility in water, the OFP diacrylate crosslinker was dissolved in dimethyl sulfoxide (DMSO) with a final concentration of 0.5 mol% in the reaction mixture. To further prevent the OFP crosslinkers from precipitation, they were added to the already degassed aqueous reaction solution at 70 °C. Due to the presence of supramolecularly bonded +C and low OFP concentrations, the μgels exhibited a more homogeneous internal structure compared to μgels with only covalently incorporated crosslinker.^[^ ^28^ ^]^ For control studies, μgels with different concentrations of +C and BIS crosslinker (0.5 mol%) were additionally synthesized (Figure S1, Supporting Information).

FTIR measurements qualitatively proved the successful incorporation of the OFP into the μgels by the emergence of enlarged signals from a C═O [*ν*_(C═O)_ = 1740 cm^−1^] vibration corresponding to the OFP esters. Successful +C incorporation was confirmed by the respective O—H [*ν*_(O—H)_ = 3300 cm^−1^] vibration (Figure S2, Supporting Information). We hypothesize that all added OFP is completely incorporated into the μgel structure since nuclear magnetic resonance (NMR) transverse relaxation experiments shown by us previously^[^ ^28^ ^]^ suggest a core corona structure of PVCL/OFP μgels. This heterogeneous radial distribution of the OFP crosslinker in the μgels is only obtained when it is more rapidly consumed than the VCL.

To further investigate the effectively incorporated content of +C into purely +C‐bonded μgels, we performed quantitative Raman spectroscopy. Therefore, we used homogeneous mixtures of defined PVCL chains and +C as calibration samples and probed the C—H vibrations of PVCL at 2928 cm^–1^ and of +C at 3065 cm^–1^ (Figures S4–S6, Supporting Information). Additionally, we performed quantitative ^1^H‐NMR using dimethyl terephthalate (DMT) as internal standard (Figures S7–S9, Supporting Information) probing the ratio of the aromatic signals of DMT at 8.08 ppm and of +C at 6.59 ppm and 6.70 ppm. In conjunction, both methods revealed that the incorporated +C fraction was higher than in the reactant feed (**Table** **1** and Figures S10 and S11, Supporting Information). Analogous measurements for control μgels crosslinked with 0.5 mol% BIS in addition to +C led to comparable results (Tables S1 and S2 and Figures S10 and S12, Supporting Information).

**Table 1:** Incorporated fraction of +C within PVCL/+C μgels determined by quantitative Raman spectroscopy and ^1^H‐NMR spectroscopy

μgel sample	+C (Raman) [mol%]	+C (NMR) [wt%]	+C (NMR) [mol%]
5 mol% +C	14.86	28.9	16.32
10 mol% +C	21.82	39.4	23.78
15 mol% +C	32.37	49.2	31.68

**Figure 2:**
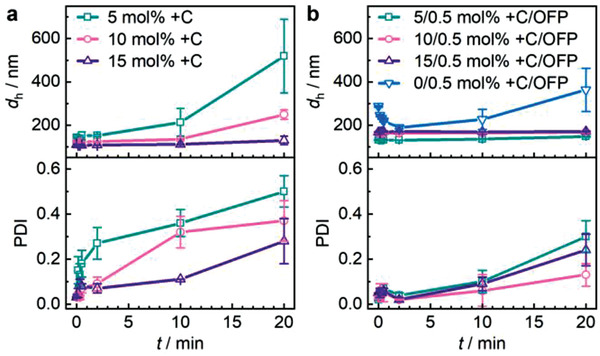
DLS of μgels with progressing sonication time a) with different +C concentrations and b) different +C concentrations in addition to OFP crosslinker (0.5 mol%). Mean values ± standard deviation (SD) from the mean. *N* = 3 independent measurements for each data point.

**Figure 3:**
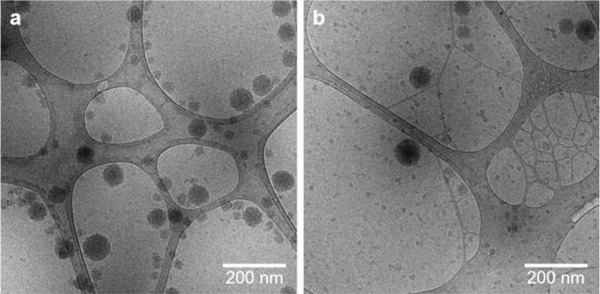
CryoTEM of μgels containing +C (15 mol%) and OFP (0.5 mol%) a) before and b) after 20 min sonication.

DLS measurements revealed that all μgels were of low dispersity with polydispersity indices (PDIs) of <0.1 and nm‐sized hydrodynamic diameters (*d*_h_) of 110–170 nm (**Figure** **2** and Figure S3, Supporting Information). Transmission electron microscopy (TEM) examinations confirmed the spherical shape of the μgels and their approximate size (**Figure** **3**). μgels observed by TEM appeared more disperse as suggested by DLS which might be related to the sample preparation and slight μgel degradation during dilution and drying caused by oxidation of +C.^[^ ^54^ ^]^

### 2.2 Mechanical Stimulation of the μgels

Shear force in solution was exerted on the μgels using an ultrasound immersion probe setup at 20 kHz ($\dot{\gamma }$ ≈ 10^9^ s^−1^).^[^ ^27^ ^]^ Over the course of the sonication process, samples were withdrawn and characterized by DLS and fluorescence spectroscopy (FS). We initially investigated μgels crosslinked only through the H‐bond interactions of +C (Figures 1b and 2a). With progressing sonication time, we observed a significant increase in hydrodynamic size suggesting the mechanical degradation of the μgels and a subsequent formation of colloidally unstable aggregates by depletion interactions.^[^ ^28^ ^]^ Yet, this PDI increase was considerably delayed and lower in extent compared to purely covalently crosslinked μgels. Higher +C content led to an enhanced resistance against shear force.

We then reasoned that μgels prepared from weak supramolecular bonds alone would dissociate rapidly under force as no strong interchain interactions were available to maintain the structural integrity of the μgels. Hence, we investigated μgels with different +C concentrations that in addition contained 0.5 mol% of the covalent OFP crosslinker (Figures 1c and 2b). Remarkably, these μgels containing two types of crosslinks, covalent and physical bonds, showed colloidal stability and only minor increases in *d*_h_ even after 20 min of ultrasonication. Notably, and as opposed to purely covalently crosslinked μgels (Figures 1a and 2b), the hydrodynamic size of H‐bonded μgels did not show an initial decrease with progressing sonication time, indicating enhanced resistance against mechanical fragmentation.

The PDI values extracted from DLS measurements (Figure 2a,b) showed that purely H‐bonded μgels were prone to a rapid increase of the PDI, indicating the formation of aggregates (Figure 2a). Conversely, the mixed covalently and H‐bonded μgels revealed a considerably delayed onset of PDI increase underlining their colloidal stability (Figure 2b). However, slight structural changes were discerned from the evolution of the PDI over sonication time. On the one hand, this was possibly rooted in minor fragmentation where the peak *d*_h_ would not be heavily altered in DLS measurements, but the PDI would reflect these changes. On the other hand, +C oxidation during sonication possibly led to a minor widening of the mesh size for some μgels without fragmentation.

The obtained results were additionally confirmed by cryoTEM (Figure 3). Even after 20 min of ultrasonication, the μgels with 15 mol% +C and 0.5 mol% OFP crosslinks appeared widely unaltered and only slight degradation was observed, which we again mainly attributed to the oxidation of +C. This is in stark contrast to purely covalently crosslinked PVCL μgels, which we reported before, where cryoTEM showed clear fragmentation after ultrasonication.^[^ ^28^ ^]^ Importantly, comparable results were obtained for μgels crosslinked with BIS instead of OFP (Figure S1, Supporting Information).

**Figure 4:**
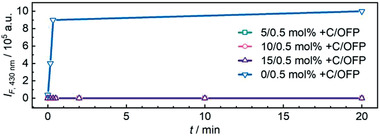
Fluorescence intensity *I*_F_ at the 0–0 transition of the OFP (*λ*_em_ = 430 nm) for μgels containing +C and OFP over the course of their sonication.

The fluorogenic OFP crosslinker then allowed us to qualitatively estimate the magnitude of force‐induced scission events of covalent bonds with progressing ultrasonication time.^[^ ^28^, ^55^ ^]^ We hypothesized that +C H‐bonds would dissipate the mechanical energy before the threshold scission force of the covalent OFP crosslinkers would be reached. Therefore, FS was performed on μgel samples over the course of the sonication experiments (Figure S13, Supporting Information). For μgels with 0.5 mol% OFP and no supramolecular bonds (Figure 1a), we observed a significant fluorescence increase in the 0–0 transition of the activated anthracene OFP (*λ*_em_ = 430 nm)^[^ ^49^, ^55^, ^56^ ^]^ already after 10 s of sonication (**Figure** **4**). Conversely, μgels with +C H‐bonds in addition to OFP (Figure 1c) showed no notable fluorescence at this wavelength thus indirectly indicating the absence of covalent bond scission events and a successful dissipation of mechanical energy by +C acting as reversible sacrificial bonds. Note that the recorded emission wavelength was chosen deliberately to avoid interference of weak +C fluorescence (vide infra) with the emission of the activated OFP. These experimental data along with DLS and TEM (Figures 2 and 3) indicated that supramolecular +C H‐bonding not only dissipated the mechanical energy protecting covalent crosslinks but also recovered after application of ultrasound maintaining the μgel structure.

Alongside, the ultrasonication of the +C‐containing μgels led to associated chemical transformations. Ultrasound in aqueous solution produces radical species which subsequently lead to oxidation reactions.^[^ ^57^ ^]^ Oxidation of +C was followed by FS where an emerging emission peak at *λ*_em_ = 437 nm was characteristic for the reversible oxidation of catechol and a peak at *λ*_em_ = 464 nm was attributed to the irreversible oxidation of the resorcinol unit.^[^ ^53^, ^54^, ^58^ ^]^ Indeed, both peaks emerged during the sonication of +C‐containing μgels (Figure S14, Supporting Information). The oxidation propensity of the μgels was dependent on +C concentration with a higher +C content decreasing the magnitude of oxidation. For μgels crosslinked with 0.5 mol% OFP and with 15 mol% +C, no such oxidation could be observed at all (Figure S13d, Supporting Information).

### 2.3 pH‐Sensitivity of the μgels

**Figure 5:**
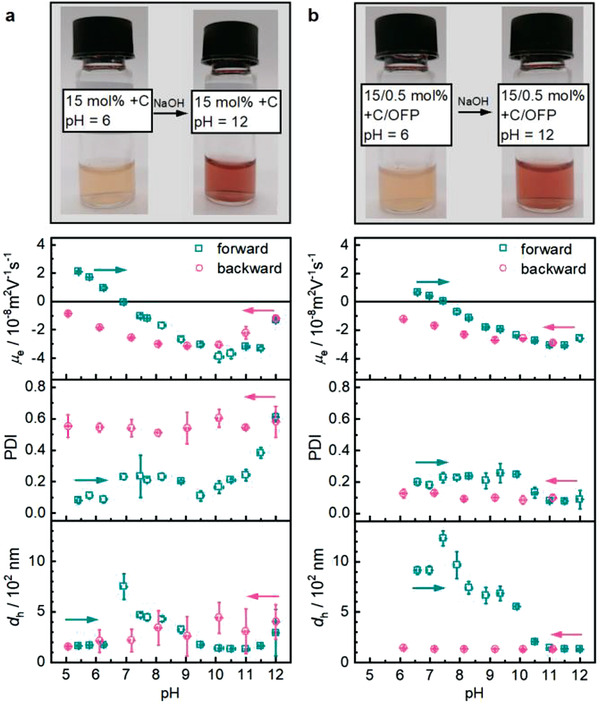
pH‐sensitivity of μgels under alkaline conditions as observed through *μ*_e_, PDI, and *d*_h_ including photographs at pH 6 and 12. a) PVCL μgels with only +C (15 mol%) and b) PVCL μgels with +C (15 mol%) and OFP (0.5 mol%). Mean values ± SD from the mean. *N* = 3 measurements at each data point.

We investigated the pH‐sensitivity of the μgels by DLS and electrophoretic light scattering using an automatic titrator. Thereby, changes in the hydrodynamic diameter, *d*_h_, PDI, and surface charge measured as *μ*_e_ were recorded at different pH. We compared μgels that either contained only supramolecular +C H‐bonds with those that contained OFP in addition to +C. At pH 6, μgel dispersions appeared slightly turbid and orange brownish (**Figure** **5**). Increasing the pH to 12, μgels containing only +C became clear, red solutions (Figure 5a) while those containing +C and OFP retained their turbid character and only adopted the red color (Figure 5b). This behavior mirrored the μgel properties under shear force: When hydroxy groups of the +C units were progressively deprotonated with increasing pH, only those μgels that were equipped with additional nonresponsive, covalent crosslinks retained their structural integrity and did not dissolve into solution.

DLS measurements showed that the *d*_h_ of all μgels increased strongly at pH 7–8. For the μgels with only +C, *d*_h_ increased to 750 nm at pH 7, and subsequently decreased again with further increase of the pH (Figure 5a). Also, the PDI slightly increased with increasing pH, proving that the degree of crosslinking was decreased by progressive deprotonation of the +C hydroxy groups. The *μ*_e_ showed that the μgels transitioned from a positive (pH <  7) over a neutral (pH = 7) to a negative (pH > 7) surface charge due to progressive +C deprotonation. After pH 12 was reached and the titration returned to pH 5, *d*_h_ and PDI showed large errors indicating μgel degradation and agglomeration.

The μgels crosslinked with OFP in addition to +C showed a maximum size of 1230 nm at pH 7.5 (Figure 5b). An increase of the PDI to 0.23 was observed at this pH indicating that temporary agglomeration of the μgels caused the increase of *d*_h_. This interpretation was supported by the *μ*_e_ which reached the isoelectric point at this pH and led to absence of electrostatic repulsion. The μgels transitioned from a positive (pH < 7.5) over a neutral (pH = 7.5) to a negative (pH > 7.5) surface charge due to progressive +C deprotonation until a constant *d*_h_ of 126 nm was reached at pH 11.5. The H‐bonds dissociated at this pH and +C was irreversibly detached from the μgels. The covalent crosslinks were not pH‐sensitive and thus remained intact. Therefore, only a partial degradation of the μgels was observed at strongly alkaline pH, which was indicated by the constant *d*_h_ upon returning to pH 6. The constant PDI around 0.1 additionally confirmed the narrow size distribution of these μgels. The *μ*_e_ values after complete detachment of +C from the μgel structure and returning to the initial pH were likely not meaningful as dissolved +C either remained in solution or partially reattached to the μgel surface at pH < 7.5.

Identical experiments were carried out for μgels with a lower +C content (5 mol%). These μgels showed similar results in terms of *d*_h_, PDI, and *μ*_e_ (Figure S15, Supporting Information). Moreover, μgels with +C (5 and 15 mol%) and with and without OFP (0.5 mol%) were investigated at acidic pH. Under these conditions +C was protonated, and the H‐bonds remained stable leading to a retained μgel structure between pH 1.5 and 6 (Figure S16, Supporting Information).

## 3 Conclusion

We here carried out the synthesis and mechanical stimulation by ultrasonication of unprecedented PVCL‐based μgels bonded with +C and crosslinked with fluorogenic OFPs. We found that μgels with supramolecular bonds alone resisted mechanical disruption better than those containing only covalent crosslinks. Importantly, a combination of supramolecular +C interchain interactions and covalent crosslinks endowed the μgels with superior mechanical resistance. While purely covalently crosslinked μgels fragmented already after several seconds of sonication, those combining supramolecular interchain interactions and covalent crosslinks resisted sonication for more than 20 min. We found that while the +C H‐bonds cleaved easily, dissipated mechanical energy, and thus protected the covalent bonds of the colloidal network as sacrificial bonds, the covalent crosslinks maintained the structural integrity of the μgels supporting the reversible re‐association of the +C H‐bonds. In addition, the presence of +C introduced pH‐sensitivity. Such mechanically resistant μgels might become useful for applications in biomedicine and engineering where shear forces are expected or for force‐actuated μgel materials.^[^ ^59^, ^60^ ^]^

## 4 Experimental Section

### Materials

AMPA (97%, Sigma‐Aldrich), BIS (99%, Sigma‐Aldrich), +C (≥98%, Sigma‐Aldrich), CTAB (≥97%, Merck Millipore), DMSO (99.7% Sigma‐Aldrich), and dialysis membranes (Zellu Trans molecular weight cut‐off 12–14 kDa, Carl Roth) were used as received. *N*‐vinylcaprolactam (VCL, 98%, Sigma‐Aldrich) was purified by vacuum distillation before use. The synthesis of the OFP crosslinker was previously described in refs. [49, 55, 56].

### Synthesis of μgels

+C‐bonded PVCL μgels were synthesized by dispersion polymerization. In a 25 mL round‐bottom flask, VCL (0.132 g, 0.948 mmol) and CTAB (0.4 mg, 0.001 mmol) were dissolved in ultrapure H_2_O (9 mL) at room temperature (r.t.) and stirred for 10 min under N_2_. Subsequently, +C was dissolved in degassed abs. EtOH and 0.5 mL were added to the reaction mixture to reach a final concentration of 5, 10, or 15 mol% with respect to VCL. The solution was heated to 70 °C in an oil bath. The polymerization was initiated by the addition of 0.5 mL AMPA solution (3.3 mg, 0.012 mmol, 24 mm in deionized H_2_O) and carried out for 2 h. After cooling, the μgels were purified by membrane dialysis in deionized H_2_O for 5 d.

For μgels additionally crosslinked with covalent bonds, 0.5 mL of OFP solution (3 mg, 0.005 mmol, 10 mm in DMSO) or 0.25 mL of BIS solution (1.6 mg, 0.01 mmol, 20 mm in H_2_O) were added to the reaction mixture to the final concentration of 0.5 mol%.

### Characterization of μgels—cryoTEM

The morphology of the μgels was studied by cryoTEM (Zeiss Libra 120TEM) at an acceleration voltage of 120 kV using an FEI Vitrobot (Model Mark IV) plunge freezing station. The samples were shock frozen in liquid N_2_, and the grids were fixed to a Gatan Model 910 cryo transfer specimen holder.

### Characterization of μgels—DLS

PDI, *d*_h_, and *µ*_e_ were determined using a Zetasizer Ultra (Malvern Instruments) with disposable 10 × 10 plastic cuvettes (DTS0012) as well as disposable folded capillary cell (DTS1070) at 25 °C. A fixed scattering angle of 175° was employed for all measurements and the μgel suspensions were diluted with ultrapure H_2_O before the measurements to result in a count rate of 100–500 kcps. The measurements for *μ*_e_ were performed with an equilibration time of 120 s at 25 °C with a 10 s pause between each repeat of 10–30 runs.

### Characterization of μgels—FS

Fluorescence intensity (*I*_F_) was measured on a Horiba Fluoromax‐4P at r.t. with an excitation wavelength of 400 nm. The data interval was 1 nm and the integration time was 0.1 s. All spectroscopic measurements were carried out with quartz cuvettes (Hellma Analytics).

### Characterization of μgels—FTIR

The measurements on freeze‐dried samples were carried out in attenuated total reflection mode on a Nexus 470 spectrometer from Thermo Nicolet at the spectral resolution of 4 cm^−1^.

### Characterization of μgels—NMR

NMR spectra were measured with a Bruker DPX‐400 FT‐NMR spectrometer at a frequency of 400 MHz with a standard pulse delay of 1 s. For each measurement the sample (10–15 mg) was dissolved in DMSO‐d_6_ (600 µL). The samples for the quantitative ^1^H‐NMR experiments were dissolved in DMSO‐d_6_ (600 µL) containing DMT (Standard for quantitative NMR, *Trace*CERT, Sigma‐Aldrich) as internal standard (8.343 mg mL^–1^) and measured with a standard pulse delay of 15 s. The recorded spectra were analyzed with the software Mestrenova (Version 12.0.1‐20560).

### Characterization of μgels—Raman Spectroscopy

Raman spectra were recorded with a Bruker RFS 100/S spectrometer equipped with a neodymium‐doped yttrium aluminum garnet laser with a wavelength of 1064 nm. Solid samples were measured with an output power of 200 mW. Each sample was measured with 1000 scans and covered a spectral range from 400 to 4000 cm^–1^ with a resolution of 4 cm^–1^. The analysis and the baseline correction of the recorded spectra were performed with the software OPUS 4.0. The Raman spectra were further processed with Origin to perform a normalization. To obtain the samples for the calibration, linear PVCL chains and +C were combined in defined ratios and dissolved in MeOH. The solvent was evaporated, and the dry and homogeneous mixtures were measured as solid powders. The μgels were measured as freeze‐dried samples.

### Characterization of μgels—Titration of μgels

The pH‐dependency of *d*_h_, PDI, and *µ*_e_ were carried out in a plastic zeta cuvette (DTS1070) using a Zetasizer Ultra equipped with an MPT‐3 Auto titrator (Malvern Instruments). The μgel dispersions (1 mL) were diluted in ultrapure H_2_O (high‐performance liquid chromatography grade, 10 mL). The pH‐values were adjusted using either aq. HCl or aq. NaOH solutions with concentrations of 0.01 m to 0.1 m. For alkaline conditions the samples were titrated from the current pH to pH 12 and back close to the starting pH. The pH was adjusted in 0.5 steps. Reaching each pH value, the sample was recirculated and equilibrated for 120 s. Then the *d*_h_ as well as the PDI were measured using an angle of 175° (backscatter) with three repetitions of 30 size runs with a duration of 1.68 s at a temperature of 25 °C. The attenuator was chosen automatically. The *µ*_e_ was also measured at each pH step with three repetitions of 10–30 runs. Between each set of runs a pause of 10 s was set. The acidic conditions were investigated in two steps. The first from the current pH to pH 2.5 and back close to the starting pH. The second from the starting pH to pH 2.5, then in 0.5 steps to a pH of 1.5 and back to pH 2.5. The measurement settings for *d*_h_, PDI, and *µ*_e_ were identical to the measurements in alkaline pH range.

### Characterization of μgels—Ultrasonication

The sonochemical irradiation experiments were carried out on a Vibra‐Cell ultrasonic processor VCX500 (Sonics and Materials) operating at 500 W in a Suslick vessel (Sonics and Materials) under an inert atmosphere. The pristine μgels (1 wt%) were diluted 20× in deionized H_2_O. 10 mL of the samples were sonicated for different periods of time (10, 20, and 30 s and 2, 10, and 20 min) while ice‐cooled, with a 13 mm probe, in the pulsed sonication mode (1 s “on” and 2 s “off”). The frequency was 20 kHz, and the amplitude was 30%.

## Conflict of Interest

The authors declare no conflict of interest.

## Supporting information

Supporting Information

## Data Availability

The data that support the findings of this study are openly available in Zenodo at https://doi.org/10.5281/zenodo.5815297, reference number [61].
