# Supplementary material for: Mechanically Resistant Poly(N‐vinylcaprolactam) Microgels with Sacrificial Supramolecular Catechin Hydrogen Bonds
Source: Adv Sci (Weinh). 2022 Feb 20;9(12):2104004. doi: 10.1002/advs.202104004 (PMC9036020; doi:10.1002/advs.202104004)
Supplement: Supplementary file 1 — Supporting Information [file ADVS-9-2104004-s001.pdf]

ADVANCED SCIENCE
Open Access

Supporting Information

for *Adv. Sci.*, DOI 10.1002/advs.202104004

Mechanically Resistant Poly(*N*-vinylcaprolactam) Microgels with Sacrificial Supramolecular Catechin Hydrogen Bonds

*Emilia Izak-Nau, Susanne Braun, Andrij Pich and Robert Göstl**

Supporting Information for

Mechanically Resistant Poly(*N*-vinylcaprolactam) Microgels with Sacrificial Supramolecular Catechin Hydrogen Bonds

*Emilia Izak-Nau, Susanne Braun, Andrij Pich, and Robert Göstl**

E. Izak-Nau, S. Braun, A. Pich, and R. Göstl
DWI – Leibniz Institute for Interactive Materials
Forckenbeckstr. 50, 52056 Aachen, Germany
E-mail: goestl@dwi.rwth-aachen.de

S. Braun, A. Pich
RWTH Aachen University, Institute of Technical and Macromolecular Chemistry
Worringerweg 1, 52074 Aachen, Germany

A. Pich
Maastricht University, Aachen Maastricht Institute for Biobased Materials (AMIBM)
Brightlands Chemelot Campus, 6167 RD Geleen, The Netherlands

Table of Contents

1. Results and Discussion 2
1.1. BIS- and +C-Crosslinked µgels 2
2. Supporting Measurements and Data 3
2.1. Fourier-Transform Infrared (FTIR) Spectroscopy 3
2.2. Dynamic Light Scattering (DLS) 3
2.3. Quantitative Raman Spectroscopy 4
2.4. Quantitative ^{1}H-Nuclear Magnetic Resonance (NMR) Spectroscopy 5
2.5. Fluorescence Spectroscopy (FS) 9
2.6. Titrations for pH-Sensitivity 10
3. References 10

1. Results and Discussion

1.1. BIS- and +C-Bonded µgels

To compare the results with regular PVCL-based µgels crosslinked with 0.5 mol% BIS instead of OFP, µgels were sonicated for the same duration. µgels crosslinked only with BIS showed a core corona structure with a more crosslinked core and a less crosslinked corona. For those µgels a fast decrease of the size was observed, because the loosely crosslinked corona was degraded by sonication. This was followed by a strong aggregation of the µgels after 2.5 min of sonication time.[1] The observed trends for the µgels crosslinked with BIS and +C were comparable to those obtained for only +C-crosslinked µgels (Figure S1). In general, the µgels appeared to be slightly more resistant to shear force than those crosslinked *via* +C only, but not as stable as the µgels crosslinked with OFP and +C. The µgels crosslinked with 0.5 mol% BIS and 15 mol% +C showed no changes in their size within a sonication time of 20 min. However, the µgels composed of 0.5 mol% BIS and 5 mol% +C as well as 10 mol% of +C were less stable than those with 0.5 mol% OFP instead of BIS. This indicated that the OFP molecules additionally increased the mechanical stability of the µgels.

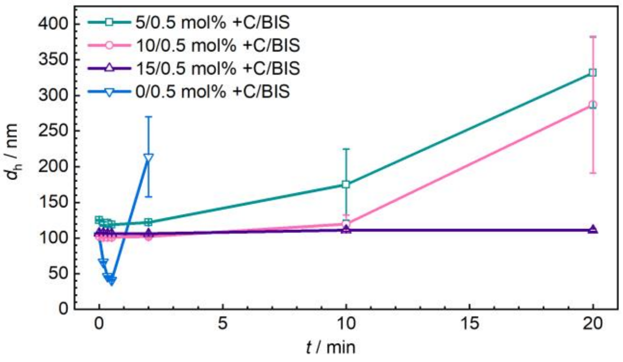

Figure S1. Hydrodynamic diameters d_h gathered from DLS intensity-weighted distributions at T = 25 °C of PVCL µgels bonded with +C (0, 5, 10, and 15 mol%) and BIS crosslinker (0.5 mol%) over the course of sonication time. Mean values ± SD from the mean. N = 3 independent measurements for each data point.

2. Supporting Measurements and Data

2.1. Fourier-Transform Infrared (FTIR) Spectroscopy

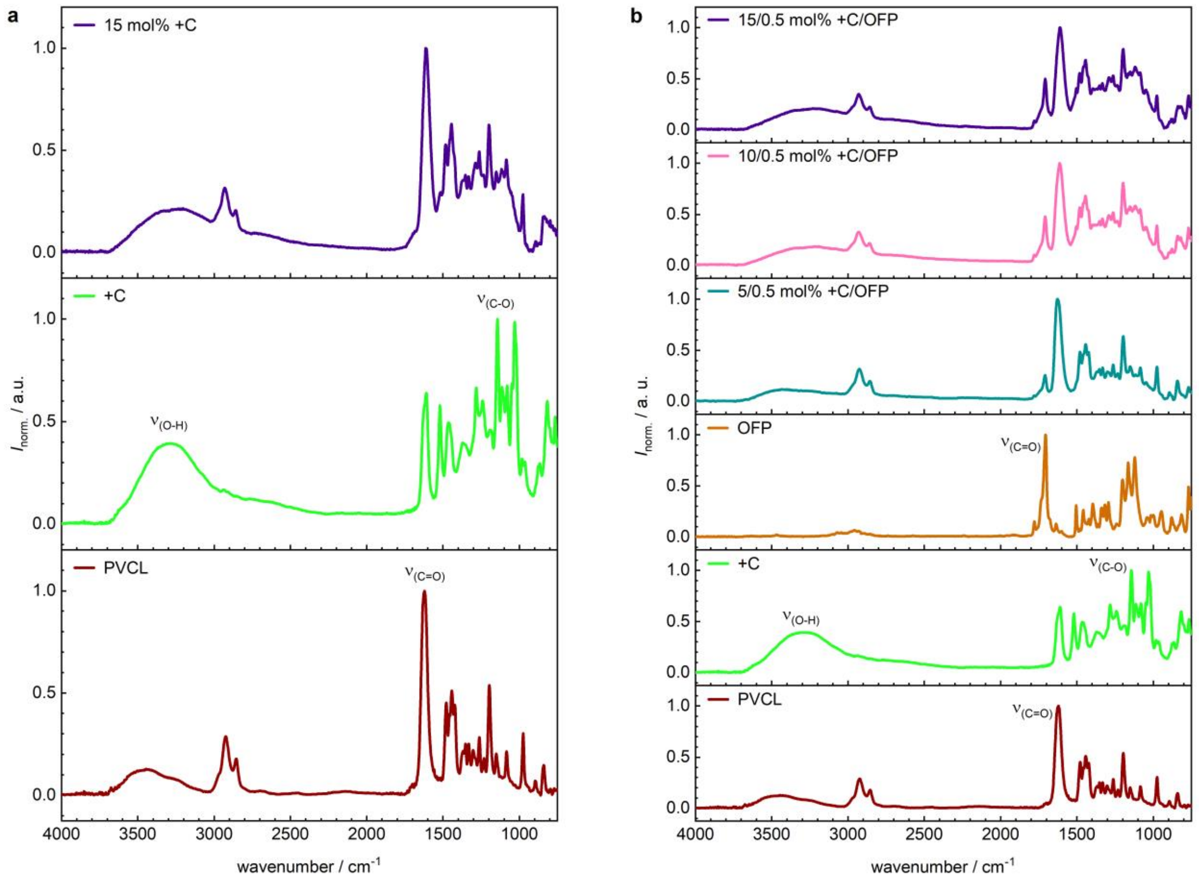

Figure S2. Stacks of FTIR spectra of µgels bonded with (a) +C and associated starting material references and (b) +C and OFP crosslinker including associated starting material references.

2.2. Dynamic Light Scattering (DLS)

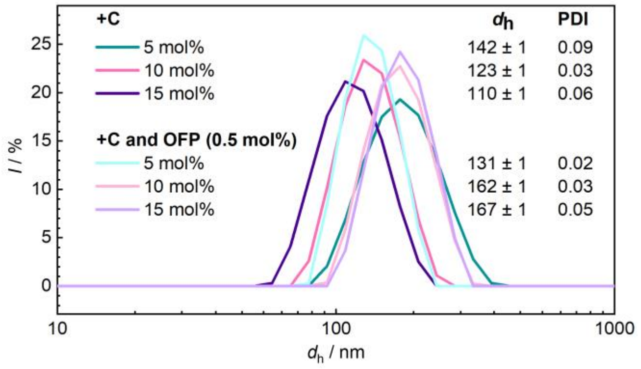

Figure S3. DLS intensity-weighted distributions at T = 25 °C of PVCL µgels bonded only with +C and those with +C together with OFP crosslinker. Mean values ± SD from the mean. N = 3 independent measurements for each data point.

2.3. Quantitative Raman Spectroscopy

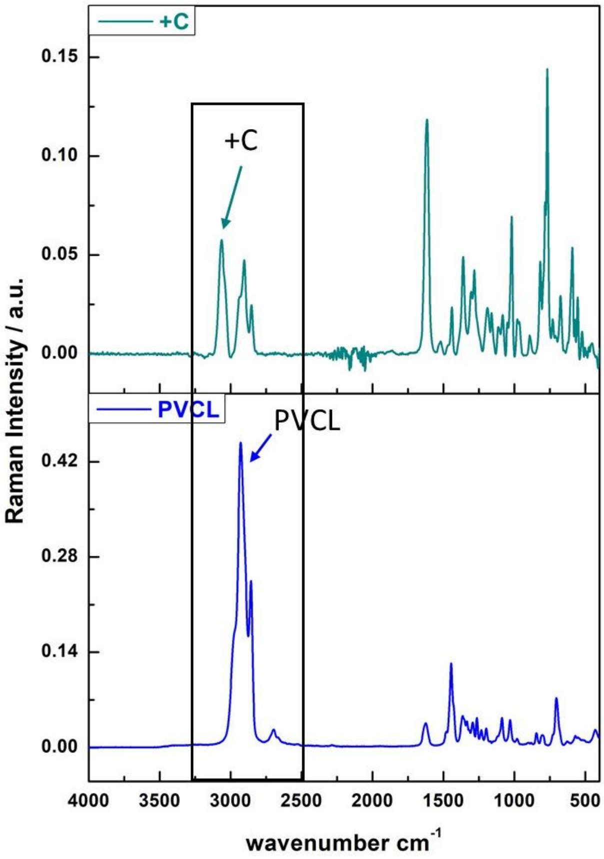

Figure S4. Raman spectra of pure calibration samples (linear PVCL chains and +C).

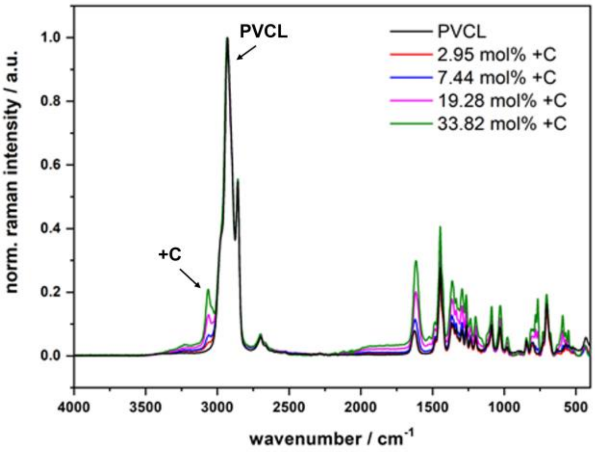

Figure S5. Raman spectra of sample mixtures of linear PVCL and defined amounts of +C to obtain a calibration curve to calculate the +C content in the µgels.

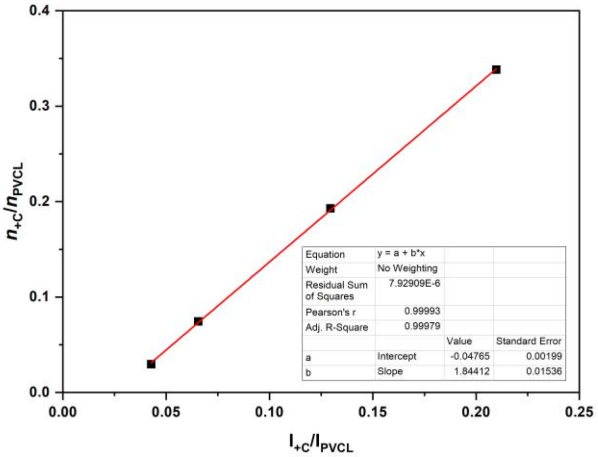

Figure S6. Calibration curve obtained by Raman spectroscopy to calculate the +C content in the µgels.

Table S1. Incorporated fraction of +C within PVCL/BIS/+C-µgels with a BIS content of 0.5 mol% determined by Raman spectroscopy.

µgel sample	+C / mol%
5/0.5 mol% +C/BIS	14.12
10/0.5 mol% +C/BIS	19.32
15/0.5 mol% +C/BIS	25.11

2.4. Quantitative ^{1}H-Nuclear Magnetic Resonance (NMR) Spectroscopy

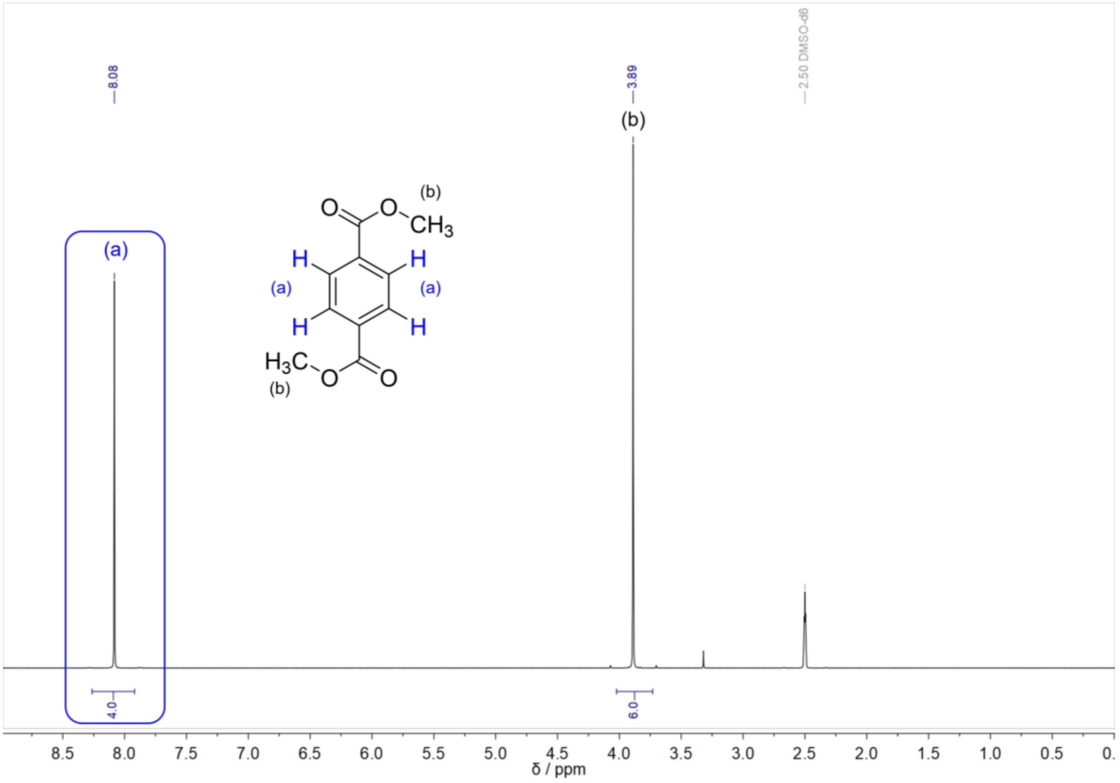

Figure S7. ^{1}H-NMR spectrum of the internal standard DMT measured in DMSO-d_6. Highlighted is the signal of the aromatic protons used for the calculation of the +C content.

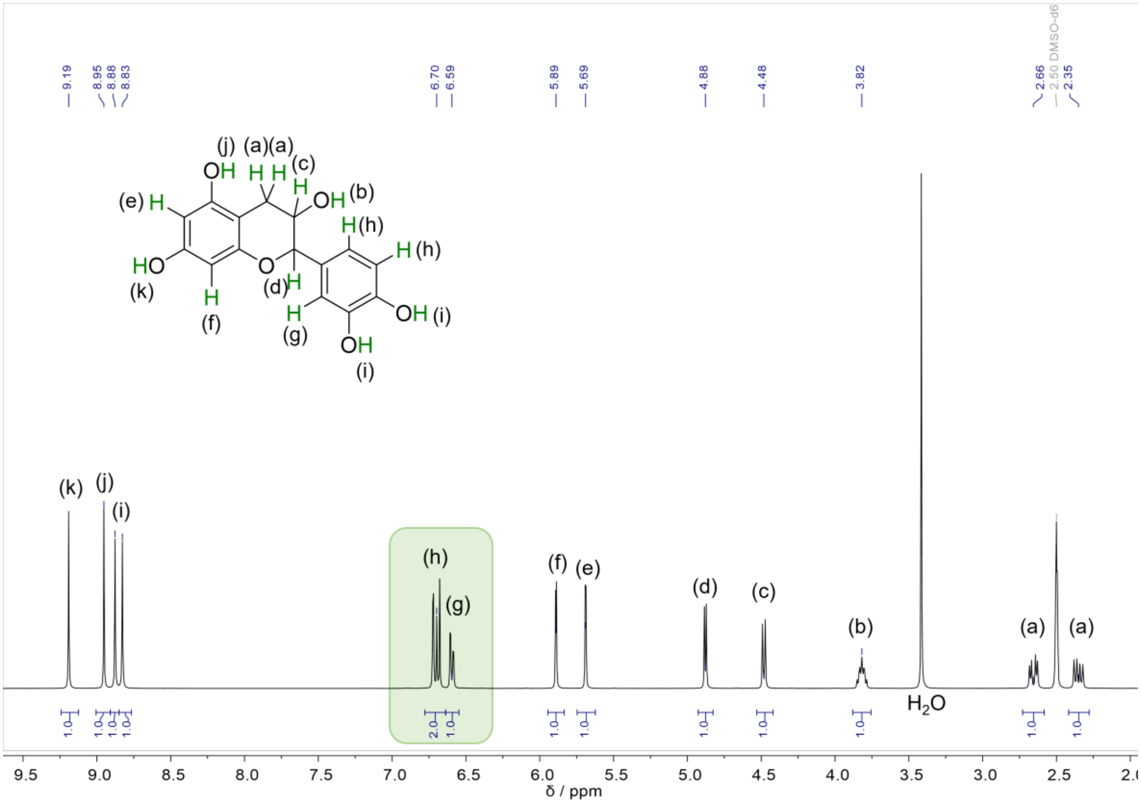

Figure S8. ^{1}H-NMR spectrum of +C measured in DMSO-d_6. Highlighted are the signals of the aromatic protons used for the calculation of the +C content.

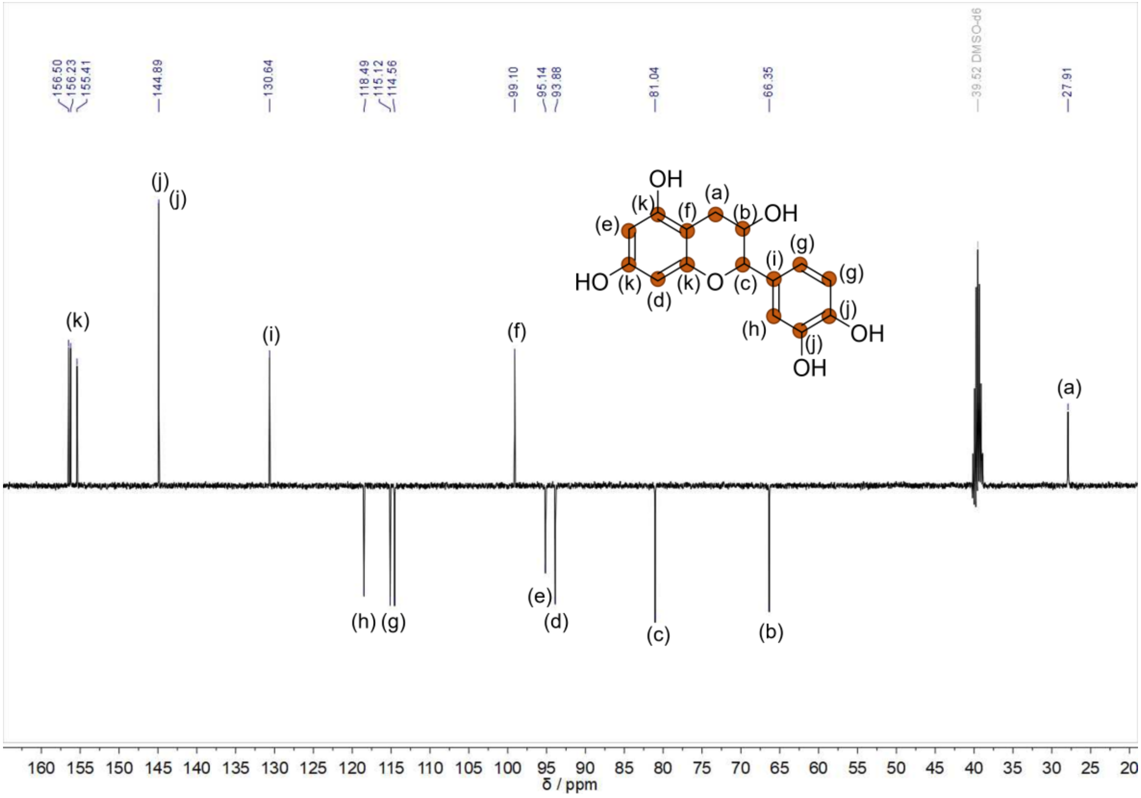

Figure S9. ^{13}C-NMR spectrum of +C measured in DMSO-d_6.

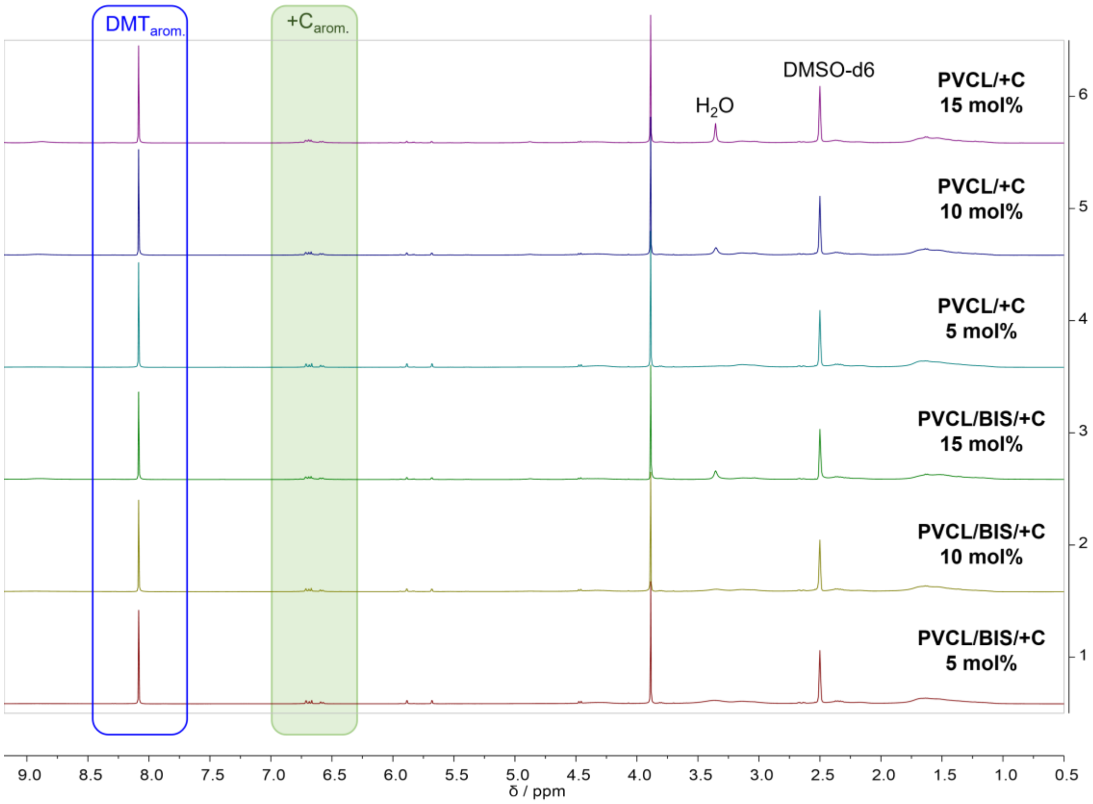

Figure S10. Stacked ^{1}H-NMR spectra of µgels bonded with supramolecular +C and µgels crosslinked with BIS and +C measured in DMSO-d_6 containing the internal standard DMT. Highlighted are the signals of the aromatic protons of the DMT and the +C used for the calculation of the crosslinker content.

Table S2. Incorporated fraction of +C within PVCL/BIS/+C µgels with a BIS content of 0.5 mol% determined by quantitative ^{1}H-NMR spectroscopy.

µgel sample	+C / wt%	+C / mol%
5/0.5 mol% +C/BIS	24.6	13.53
10/0.5 mol% +C/BIS	32.5	18.74
15/0.5 mol% +C/BIS	35.4	20.84

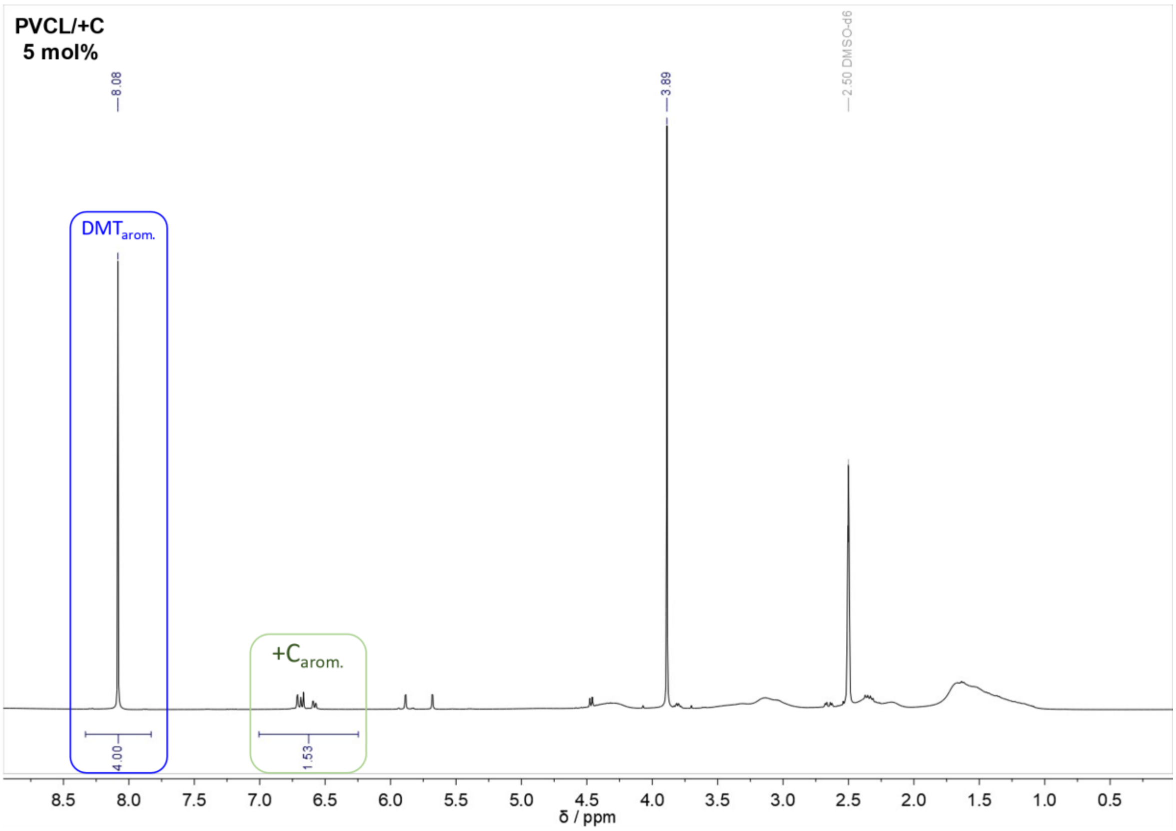

Figure S11. Exemplary ^{1}H-NMR spectrum of PVCL/+C µgels measured in DMSO-d_6 containing the internal standard DMT. Highlighted are the signals of the aromatic protons of DMT and +C used for the calculation of the +C content.

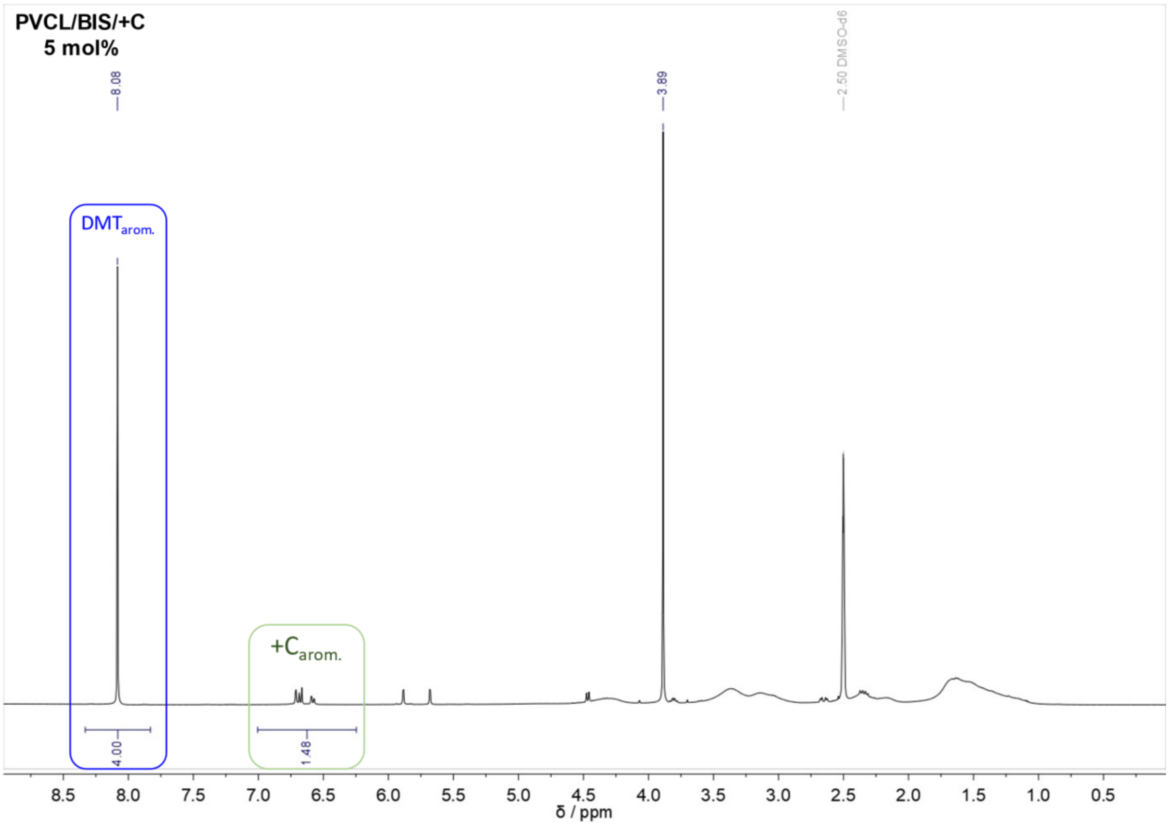

Figure S12. Exemplary ^{1}H-NMR spectrum of PVCL/BIS/+C µgels measured in DMSO-d_6 containing the internal standard DMT. Highlighted are the signals of the aromatic protons of DMT and +C used for the calculation of the physical crosslinker content.

2.5. Fluorescence Spectroscopy (FS)

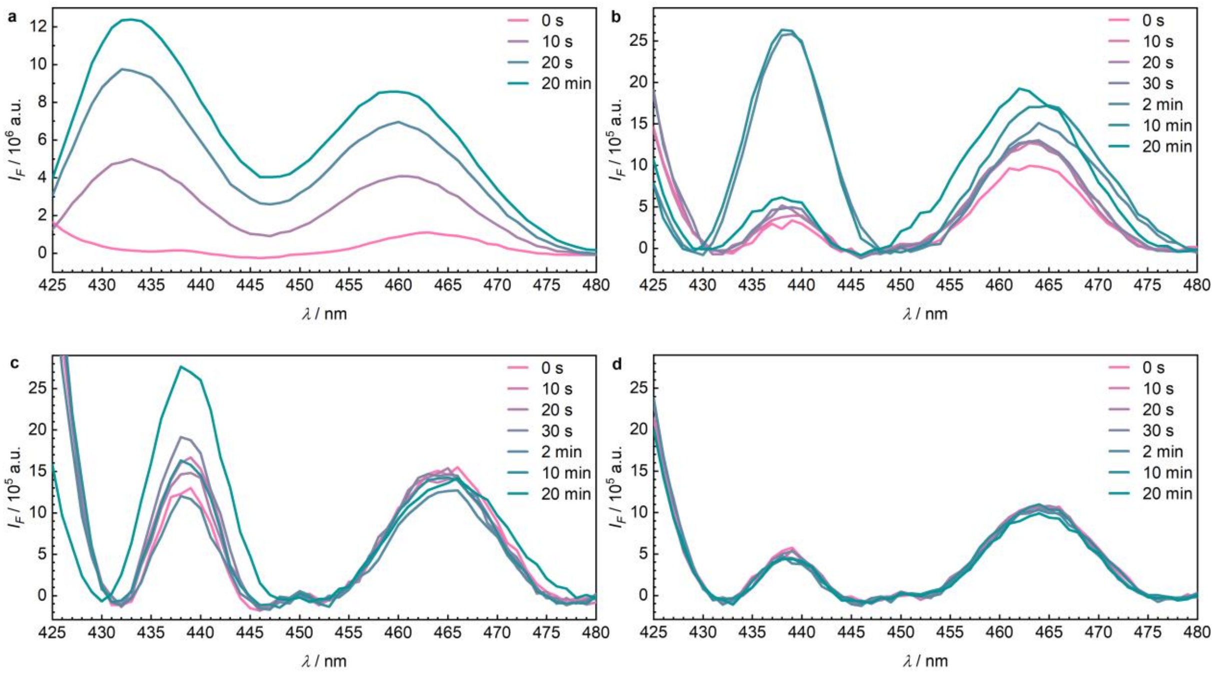

Figure S13. Fluorescence intensity I_F for µgels bonded with +C and OFP crosslinker (0.5 mol%) over the course of the sonication. (a) +C (0 mol%), (b) +C (5 mol%), (c) +C (10 mol%), and (d) +C (15 mol%).

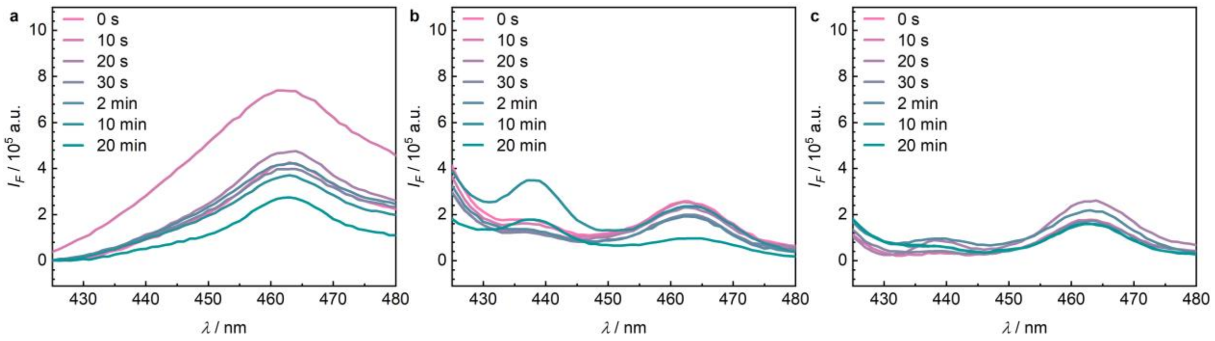

Figure S14. Fluorescence intensity I_F for µgels bonded with +C over the course of the sonication. (a) 5 mol%, (b) 10 mol%, and (c) 15 mol%.

2.6. Titrations for pH-Sensitivity

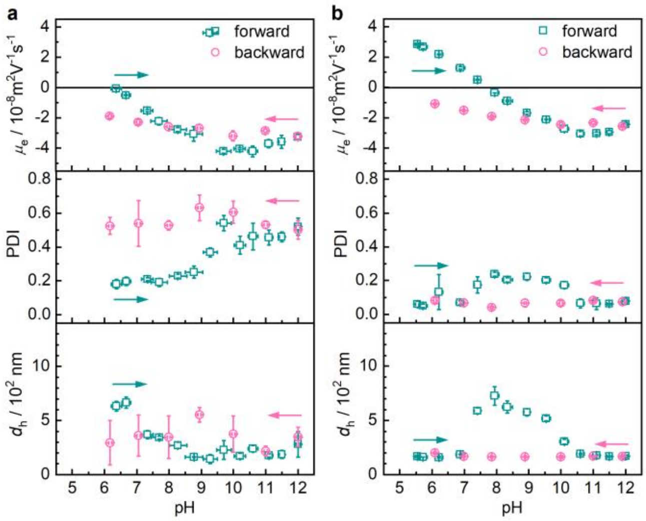

Figure S15. pH-sensitivity of µgels under alkaline conditions. d_h, PDI, and μ_e for µgels crosslinked with (a) +C (5 mol%) and (b) +C (5 mol%) and OFP (0.5 mol%).

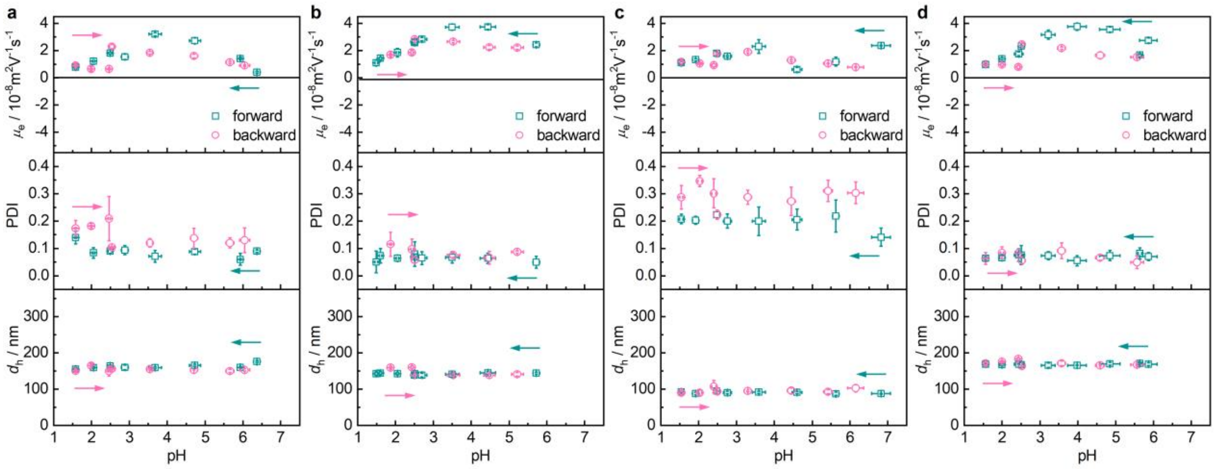

Figure S16. pH-sensitivity of µgels under acidic conditions. d_h, PDI, and μ_e for µgels crosslinked with (a) +C (15 mol%), (b) +C (15 mol%) and OFP (0.5 mol%), (c) +C (5 mol%), and (d) +C (5 mol%) and OFP (0.5 mol%).

3. References

[1] E. Izak-Nau, D. E. Demco, S. Braun, C. Baumann, A. Pich, R. Göstl, *ACS Appl. Polym. Mater.* **2020**, *2*, 1682-1691.
